# Tension Force Estimation in Axially Loaded Members Using Wearable Piezoelectric Interface Technique

**DOI:** 10.3390/s19010047

**Published:** 2018-12-22

**Authors:** Joo-Young Ryu, Thanh-Canh Huynh, Jeong-Tae Kim

**Affiliations:** Department of Ocean Engineering, Pukyong National University, Busan 48723, Korea; cozy1028@nate.com (J.-Y.R.); ce.huynh@gmail.com (T.-C.H.)

**Keywords:** axial member, tension force, wearable technology, piezoelectric interface, impedance signatures, impedance method

## Abstract

Force changes in axially loaded members can be monitored by quantifying variations in impedance signatures. However, statistical damage metrics, which are not physically related to the axial load, often lead to difficulties in accurately estimating the amount of axial force changes. Inspired by the wearable technology, this study proposes a novel wearable piezoelectric interface that can be used to monitor and quantitatively estimate the force changes in axial members. Firstly, an impedance-based force estimation method was developed for axially loaded members. The estimation was based on the relationship between the axial force level and the peak frequencies of impedance signatures, which were obtained from the wearable piezoelectric interface. The estimation of the load transfer capability from the axial member to the wearable interface was found to be an important factor for the accurate prediction of axial force. Secondly, a prototype of the wearable piezoelectric interface was designed to be easily fitted into existing axial members. Finally, the feasibility of the proposed technique was established by assessing tension force changes in a numerical model of an axially loaded cylindrical member and a lab-scale model of a prestressed cable structure.

## 1. Introduction

In civil structures, axially loaded members, such as columns, connecting rods, cables, and trusses, are common elements that are subjected to only tension or compression. During their service life, these members may be exposed to repeated overloads and severe ambient conditions that could induce local failures, leading to a reduction in their future load-carrying capacities. Thus, in-situ loading status monitoring of these members is of significant interest in evaluating the operational safety of a whole structure.

There have been several research attempts to monitor the tension of axial members. Since the 1980s, vibration-based structural health monitoring (SHM) has been widely adopted to estimate the axial force in cable and beam-like structures [1,2,3,4,5]. The vibration technique is relatively simple to perform and can provide reliable axial load estimation, but it requires measurements of vibration responses that should be generated by sufficient external excitations. Another simple way is to estimate the axial load by measuring the strain of axial members. Electrical strain gauges are low cost but do not own a natural reference point, which may cause a significant error under long-term monitoring. Therefore, some researchers have used optical strain sensors (e.g., fiber Bragg grating (FBG) sensors) for force monitoring of axial members, such as cable structures [6,7]. FBG sensors have high precision, self-referencing capability, and long service life, but they need to be embedded into the cables through a complicated and expensive manufacturing process [8,9].

In recent years, impedance-based SHM has emerged as a promising approach for integrity assessment of civil infrastructures. The impedance technique is enabled by a piezoelectric sensor, e.g., lead zirconate titanate (PZT), which is surface-bonded to a monitored structure to measure electromechanical (EM) impedance. Because the measured EM impedance contains local dynamic features of the monitored structure, impedance variations can be used as a local, sensitive signal to detect damage occurrence. Major advantages of the impedance technique include self-excitation and examination without external loads, cost-effective sensors and cheap data acquisition system, high sensitivity to small damage sizes, and autonomous and real-time monitoring. Various experimental and numerical studies in lab-scale and on real structures have demonstrated the practicality of the impedance-based SHM technique [10,11,12,13,14,15,16,17].

However, the direct attachment method of the PZT often influences the appearance of the monitored structure and causes difficulties in reconfiguration of the sensor in necessary cases [18,19]. An additional important issue is the repeatability of impedance signatures, which is dependent on the structural condition of the bonding layer and the surficial condition of the target surface [20]. To overcome these issues, Annamdas et al. [18] developed an indirect attachment technique using a portable structure embedded with PZTs to indirectly acquire impedance signatures from a host structure. To enhance the sensitivity of impedance signatures and predetermine an effective frequency band, Huynh and Kim [21] proposed a portable PZT interface technique for tendon-anchorage monitoring. The interface technique was then modified using magnetic blocks for attaching the PZT-embedded portable interface to a host structure [19]. Recently, Wang et al. [22] designed a wearable sensor device comprising PZTs for health monitoring of bolted joints. These studies demonstrate the promising value of wearable devices for impedance-based SHM.

Owing to its promising advantages, many researchers have used the impedance-based technique to monitor the tension force in axial members, such as prestressing strands [23,24,25], in steel rods [26], and in rock bolts [27]. However, the studies had drawbacks that hindered them from being used widely. One of the important problems is the difficulty in quantitatively estimating the damage severity and the change in structural parameters using well-known statistical damage metrics such as root mean square deviation (RMSD) or cross-correlation deviation (CCD) because these metrics are not physically related to the mechanical properties. Several research attempts have been made to deal with this issue. Ritdumrongkul et al. [28] used model updating to correlate the measured impedance signatures with the analytical signatures for quantitative torque monitoring of bolted joints. Lu et al. [20,29] proposed a PZT-embedded smart probe for strength development monitoring of cementitious materials and developed an analytical impedance-based model using the resonant frequencies of the smart probe for strength estimation. Huynh et al. [30] developed a method using peak frequencies of the portable PZT interface and a model-updating algorithm to assess the contact parameters of tendon anchorage.

Inspired by the wearable technology, this study proposes a novel piezoelectric interface-based impedance method that can be used to monitor and quantitatively estimate the force changes in axial members. Firstly, an impedance-based force estimation model was designed for axially loaded members. The estimation was based on the relationship between the axial force level and the peak frequencies of impedance signatures, which were obtained from the wearable piezoelectric interface. The estimation of the load transfer capability from the axial member to the wearable interface was found to be an important factor for the accurate prediction of axial force. Secondly, a prototype of the wearable piezoelectric interface was designed to be easily fitted into existing axial members. Finally, the feasibility of the proposed technique was established by assessing tension force changes in the numerical model of an axially loaded cylindrical member and a lab-scale model of a prestressed cable structure.

## 2. Impedance-Based Axial Load Estimation Method

### 2.1. Schematic of the Method

**Figure 1:**
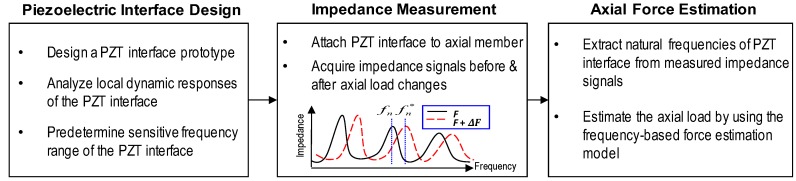
Axial force estimation method using impedance measurement.

Previous works have proven that the peaks of impedance signatures represent the modal frequencies of the PZT-driven system [31,32]. This suggests that the natural frequencies of a host structure can be extracted from the resonant peaks of impedance signatures [30,33,34]. By utilizing this advantage, we developed an axial force estimation method using the impedance measurement, as shown in Figure 1. The idea was to estimate the axial load using the natural frequencies of a PZT interface structure, which were obtained from impedance measurements. The proposed method was performed through three main steps. In Step 1, a piezoelectric interface prototype was designed. Then, local dynamic characteristics of the interface were analyzed to predetermine the sensitive frequency range of impedance responses that contain the modal frequencies. In Step 2, the PZT interface prototype was attached to an axially loaded member. The impedance signatures of the PZT interface were measured before and after load change events. In Step 3, the natural frequencies of the PZT interface were extracted from the measured impedance signatures. Then, the axial force of the monitored structure was estimated using a frequency-based force estimation model.

### 2.2. Axial Force Estimation Model

**Figure 2:**
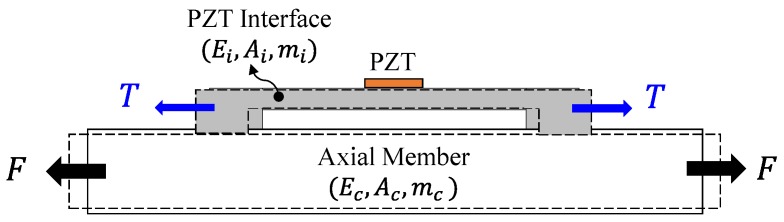
Piezoelectric interface attached to an axial member under tension.

The axial load estimation method was proposed on the basis of the piezoelectric interface technique, which was originally developed to acquire sensitive impedance signatures [21]. The piezoelectric interface is typically a beam-like structure that is embedded with a PZT sensor at the middle. To monitor the axial force, a PZT interface was attached to an axially loaded member, as schematized in Figure 2. A tension force F applied to the axial member will induce an external axial load T into the PZT interface. The variation of F will result in the alternation of T, leading to the shift in the modal frequency of the interface. By catching the frequency shift in the impedance signatures, it is possible to detect and estimate the variation of the applied force F.

The governing differential equation of the beam-like interface under axial force T is given as follows [35]:

(1) $$\frac{\partial^{4}\nu \left(x,t\right)}{\partial x^{4}}-\frac{T}{E_{i}I_{i}}\frac{\partial^{2}\nu \left(x,t\right)}{\partial x^{2}}+\frac{m_{i}}{E_{i}I_{i}}\frac{\partial^{2}\nu \left(x,t\right)}{\partial t^{2}}=0$$

where $m_{i}$ is mass per unit area; $E_{i}$ and $I_{i}$ are Young’s modulus and moment of inertia of the interface, respectively. The n^th^ natural frequency of the interface that is dependent on its boundary condition can be determined as follows:

(2) $$f_{n}=\frac{C_{n}}{2{\pi L}_{i}{}^{2}}\sqrt{1\eta +\frac{{\mathrm{TL}}_{i}{}^{2}}{E_{i}I_{i}\pi^{2}}\frac{C_{1}}{C_{n}}}\sqrt{\frac{E_{i}I_{i}}{m_{i}}}$$

where η = ¼ for a fixed-fixed (F-F) interface, and η = 1 for a pined-pined (P-P) interface. C_1_ and C_n_ are nondimensional frequencies of the 1^st^ mode and the n^th^ mode of the interface, respectively, as listed in Table 1 [35], and $L_{i}$ is the length of the interface’s flexible part.

When the monitored axial member experiences a force change ΔF = F^*^ − F, the interface experiences a corresponding shift in its axial load ΔT = T^*^ − T, and the natural frequency of the interface is turned from $f_{n}$ to $f_{n}^{*}$. By introducing the change in square frequencies $\Delta \left(f_{n}^{2}\right)=f_{n}^{*}{}^{2}-f_{n}^{2}$ into Equation (2), a formula representing the physical relationship between the force change in the interface ΔT, and the frequency change is obtained as follows:

(3) $$\Delta T=\frac{4m_{i}\pi^{4}L_{i}{}^{2}}{\eta C_{1}C_{n}}\Delta \left(f_{n}^{2}\right)$$

Under the applied load F, the strain of the axial member is transferred to the wearable PZT interface. Therefore, the load change ΔF can be easily correlated with ΔT as follows:

(4) $$\Delta F=\alpha \frac{E_{c}A_{c}}{E_{i}A_{i}}\Delta T$$

where E_c_ and A_c_ are Young’s modulus and area of the axial member, respectively; E_i_ and A_i_ signify Young’s modulus and area of the interface, respectively. The term α is a load transfer capability factor considering the attachment strength of the interface device to the axial member. A larger α suggests a lower capability of load transfer to the interface. The value of α is close to 1 when the interface is perfectly bonded to the structure or the applied load is completely transferred to the interface.

By substituting Equation (4) into Equation (3) and considering NM vibration modes of the interface, the tension force change in the monitored structure can be obtained:

(5) $$\Delta F=\alpha \frac{E_{c}A_{c}}{E_{i}A_{i}}\frac{4m_{i}\pi^{4}L^{2}}{\eta C_{1}C_{n}}\frac{1}{\mathrm{NM}}\overset{\mathrm{NM}}{\underset{n=1}{\sum }}\Delta \left(f_{n}^{2}\right)$$

Using Equation (5), the load changes in an axially loaded member can be easily estimated from the natural frequencies of the piezoelectric interface. 

## 3. Design of Wearable Piezoelectric Interface for Axial Cylindrical Structure

### 3.1. Conceptual Design

**Figure 3:**
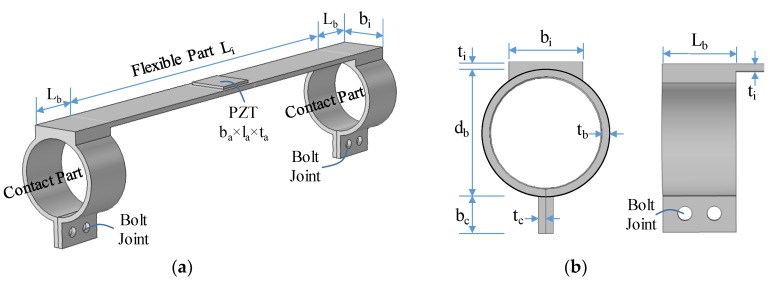
Design of wearable piezoelectric interface for cylindrical structure: (**a**) wearable lead zirconate titanate (PZT) interface; (**b**) contact part.

A novel wearable PZT interface was developed to monitor the tension force in axial cylindrical structures, as shown in Figure 3. The device has a beam-like flexible part at the middle and two outside contacting parts. The flexible part has a thin and long beam to provide the free vibration of the PZT sensor and to enhance the effect of the tension force on the natural frequencies. The two outside parts use two hoops with clamping mechanism to maximize the attachment capability of the interface to a target structure. The clamping mechanism is controlled by bolt joints, which allow the interface to be easily attached to and detached from the structure, thus enabling quick installation in the field. 

The design parameters of the interface device are defined in Figure 3. Briefly, the flexible part has width b_i_, length L_i_, and thickness t_i_; the hoop of the contact part has outer diameter d_b_, length L_b_, and thickness t_b_; and the PZT sensor has width b_a_, length l_a_, and thickness t_a_. The dimensional parameters of the hoop should be designed to match with the dimension of the axial structure. In order to minimize the effect of the PZT sensor on the natural frequency of the interface, the PZT’s dimensions should be thin enough so that its mass is ignorable compared to the mass of the flexible part of the interface. 

### 3.2. Predetermination of Sensitive Frequency Range

The sensitive frequency band of impedance signatures obtained from the interface device is decided by its modal frequencies. Thus, to identify the sensitive frequency range containing modal frequencies, the local dynamic responses of the wearable PZT interface should be analyzed in advance. 

#### 3.2.1. Finite Element Modeling

**Figure 4:**
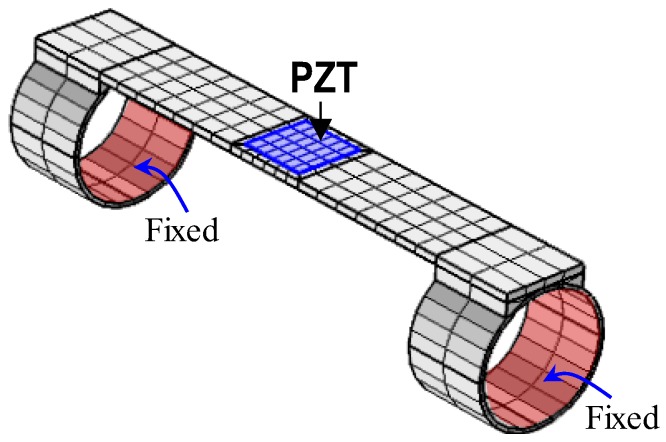
Finite element modeling of wearable PZT interface.

An example design of the wearable interface was selected with the following dimensional parameters: b_i_ × L_i_ × t_i_ = 10 × 50 × 1 mm for the flexible part, d_b_ × L_b_ × t_b_ = 16.2 × 10 × 1 mm for the hoop part (or contact part), and b_a_ × l_a_ × t_a_ = 8 × 8 × 0.127 mm for the PZT patch. A finite element (FE) model of the PZT interface was established using a commercial program, COMSOL Multiphysics, as shown in Figure 4. For simplification, the bolted joints were not simulated. A previous study by Islam and Huang [36] showed that when the host structure undergoes flexural deformations, the effect of bonding layer on the resonant frequencies is slight and can be negligible. Thus, the bonding layer between the PZT and the flexible part was not modeled. The fixed boundary condition was applied to the inner surfaces of contacting hoops of the PZT interface, see Figure 4. 

The FE model was discretized by 3D solid elements. The meshing includes 24 elements for the PZT patch and 440 elements for the interface body. Steel was used for the interface body, and PZT-5A was used for the piezoelectric sensor. The material properties of the interface body and the PZT patch are listed in Table 2 and Table 3, respectively. Two modules of solid mechanics and piezoelectric devices were coupled to model the impedance responses of the piezoelectric interface. For acquiring the electromechanical impedance, a harmonic excitation voltage with amplitude of 1V was applied to the top surface of the PZT patch, and the bottom was set as the ground electrode.

#### 3.2.2. Sensitive Frequency Range

**Figure 5:**
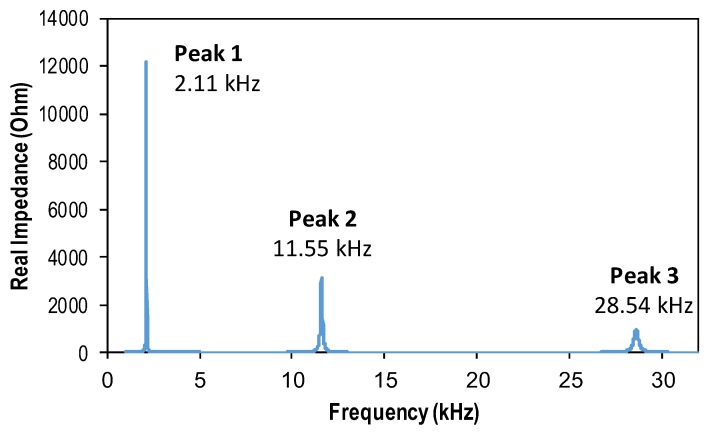
Sensitive frequency range of wearable PZT interface.

**Figure 6:**
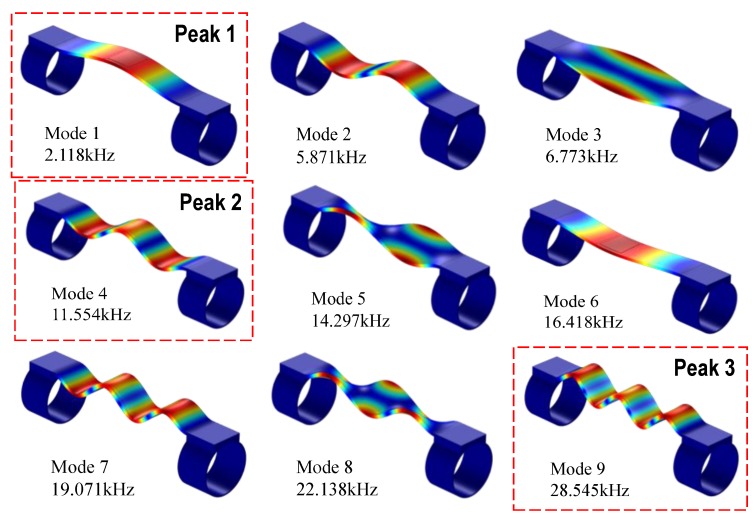
Nine modal shapes of wearable PZT interface.

The impedance response of the wearable PZT interface was analyzed under the swept frequency of 1–32 kHz. The real part of impedance was plotted in log-scale against the swept frequency, as shown in Figure 5. The figure shows the first, second, and third peak frequencies at 2.11 kHz, 11.55 kHz, and 28.54 kHz, respectively. For comparison, the modal analysis of the wearable PZT interface was also conducted. The first nine mode shapes representing the local vibrations of the interface were obtained as shown in Figure 6. Among them, Modes 1, 2, 4, 7, and 9 are longitudinal bending motions; mode 6 is a lateral bending motion; and modes 3, 5, and 8 are longitudinal twist motions.

Matching the peak frequencies of the impedance with the modal analysis result, it was shown that the first bending motion (Mode 1 at 2.12 kHz) was identical to the first impedance peak (Peak 1 at 2.11 kHz), the third bending motion (Mode 4 at 11.55 kHz) was consistent with the second impedance peak (Peak 2 at 11.55 kHz), and the fifth bending motion (Mode 9 at 28.55 kHz) agreed well with the third impedance peak (Peak 3 at 28.54 kHz). It should be noted that the PZT sensor was located at the middle of the interface, thus its ability to excite the modal motions with modal nodes at the sensor’s location (Modes 2, 3, 5, 7, 8) was very low. Moreover, under the harmonic voltage, the PZT was not bent in the lateral direction, so the ability to excite the lateral bending motion (Mode 6) was minimal. As a result, these modal motions were absent from the impedance signatures.

## 4. Numerical Evaluation of Finite Element Model of Axial Cylindrical Member

### 4.1. Finite Element Modeling of Axial Cylindrical Member with Wearable PZT Interface

**Figure 7:**
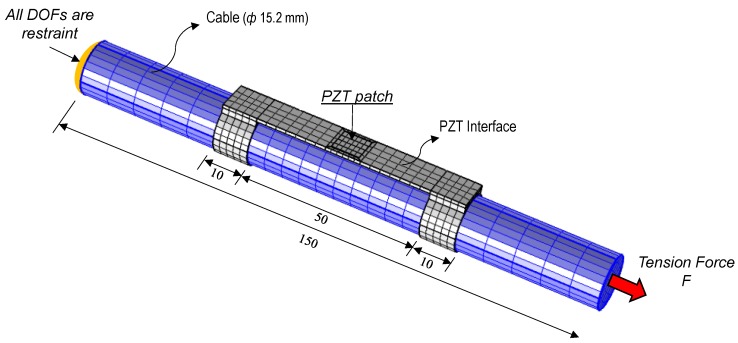
Finite element modeling of the axial cylindrical member with PZT interface (unit: mm).

To evaluate the numerical feasibility of the proposed wearable piezoelectric interface, an FE model of an axially loaded cylindrical structure equipped with a wearable PZT interface was established using COMSOL Multiphysics, as shown in Figure 7. The target structure with a diameter of 15.2 mm is made of steel, which is commonly used in practice. As only the effect of axial force was considered in the FE model, a 150 mm segment of the axial member was simulated to reduce the time and computational costs. The wearable PZT interface designed in Section 3.2 was used to acquire the impedance signatures.

The fixed boundary condition was applied at one end of the axial member, and the tension force F was introduced at the other end, as shown in Figure 7. The FE model was discretized by 3D solid elements. The meshing includes 24 elements for the PZT patch, 440 elements for the interface body, and 1080 elements for the cylindrical structure. The material properties of the cylindrical structure are the same as those of the interface body, as listed in Table 2.

### 4.2. Numerical Impedance Responses of Wearable PZT Interface

**Figure 8:**
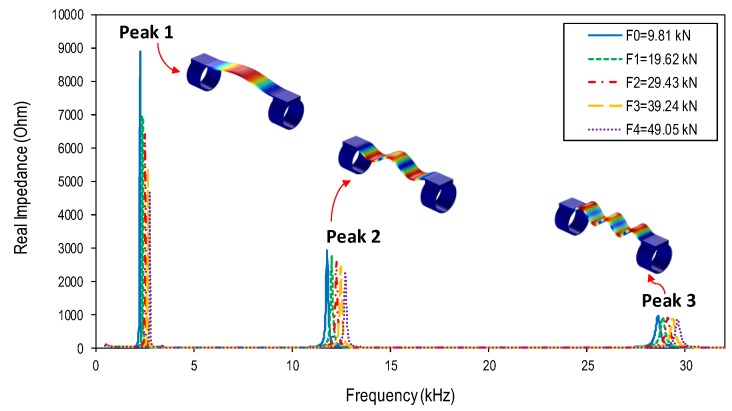
Numerical impedance signatures of wearable PZT interface under different tension forces.

Five cases of the tension force (F0–F4) were introduced into the test structure, as listed in Table 4. From the analysis in Section 3.2, the sensitive frequency range that contains the modal frequencies of the PZT interface was 1–32 kHz. Therefore, the impedance signatures in 1–32 kHz were numerically acquired from the PZT interface under the five cases of tension forces, as shown in Figure 8. As identified previously, the first impedance peak (Peak 1) corresponded to the first bending mode, the second impedance peak (Peak 2) corresponded to the third bending mode, and the third impedance peak (Peak 3) was identical to the fifth bending mode of the PZT interface.

**Figure 9:**
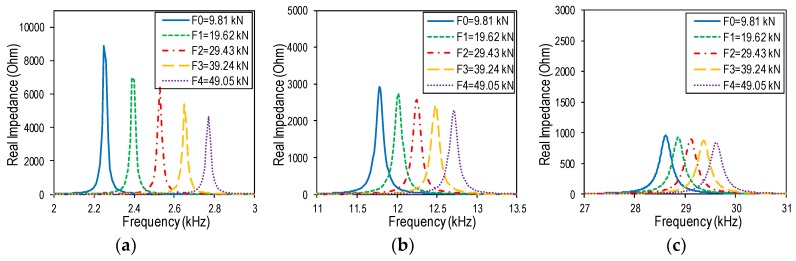
Shifts in numerical impedance peaks due to tension force changes: (**a**) Peak 1; (**b**) Peak 2; (**c**) Peak 3.

Three frequency bands containing Peaks 1–3 were zoomed in Figure 9. The figure shows that the three impedance peaks were sensitively shifted to the right when the tension force went up from F0 to F4. The increased peak frequency suggested the increment in the structural stiffness of the steel member along with the increased tension force. When the tension force was increased from F0 = 9.81 kN to F4 = 49.05 kN, the peak frequency increased gradually from 2.25 kHz to 2.77 kHz (520 Hz change) for Peak 1, from 11.78 kHz to 12.71 kHz (930 Hz change) for Peak 2, and from 28.61 kHz to 29.62 kHz (1010 Hz change) for Peak 3. The result demonstrated that the proposed wearable PZT interface can be used to monitor the axial load change in an axially loaded member via tracking shifts in the impedance peaks.

**Figure 10:**
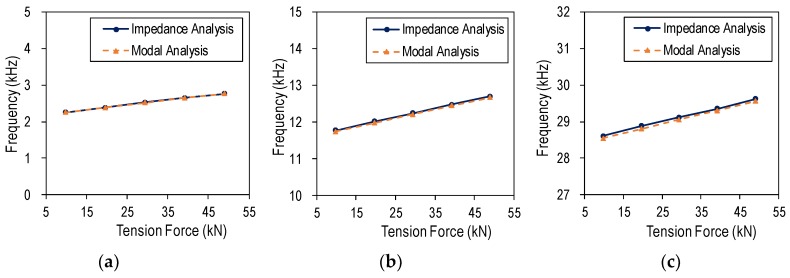
Natural frequencies of wearable PZT interface: numerical impedance analysis vs. modal analysis: (**a**) Peak 1; (**b**) Peak 2; (**c**) Peak 3.

The modal frequencies of the PZT interface are compared for the impedance analysis and the modal analysis in Figure 10. The comparison confirmed that the modal frequencies of the wearable PZT interface can be accurately obtained from the impedance measurement.

### 4.3. Estimation of Tension Force Changes in Axial Cylindrical Member

#### 4.3.1. Analytical Model of Wearable PZT Interface

**Figure 11:**
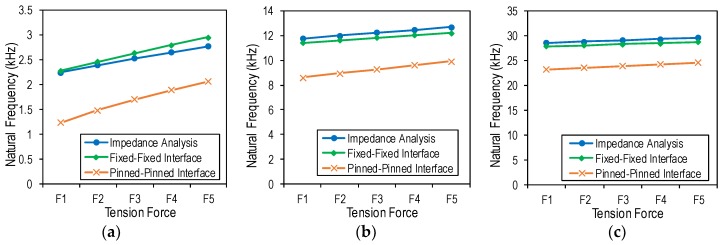
Natural frequencies of wearable PZT interface: numerical impedance analysis vs. analytical solutions (Equation (2)): (**a**) Peak 1; (**b**) Peak 2; (**c**) Peak 3.

The analytical model of the PZT interface should be determined in order to use a correct prediction formula of the tension force. For this purpose, the natural frequencies of the PZT interface from the analytical solutions (Equation 2) were compared with the impedance analysis results, as shown in Figure 11 and listed in Table 5. It can be seen that the F-F interface model showed similar natural frequencies with the impedance analysis, while the P-P interface model showed lower values. The results suggested that the analytical model of the PZT interface should have F-F boundary conditions.

#### 4.3.2. Monitoring of Tension Force Change Using Statistical Damage Metric

To monitor the tension force change, the well-known RMSD damage metric is commonly used. The metric is based on statistically quantifying the difference between the impedance signatures of damage states and the signature of the pristine state as follows:

(6) $$\mathrm{RMSD}=\sqrt{\sum_{i=1}^{N}{\left[Z^{*}(\omega_{i})-Z(\omega_{i})\right]}^{2}/\sum_{i=1}^{N}{\left[Z(\omega_{i})\right]}^{2}}$$

where $Z\left(\omega_{i}\right)$ and $Z^{*}\left(\omega_{i}\right)$ signify the impedance responses at the i^th^ frequency before and after a damage event, respectively, and N denotes the number of swept frequencies. 

**Figure 12:**
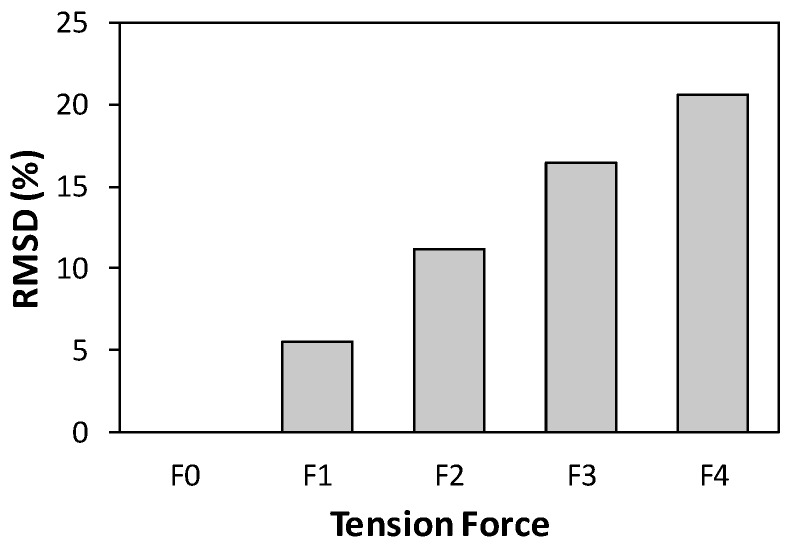
Monitoring of tension force changes in axial cylindrical member using root mean square deviation (RMSD) index.

Figure 12 shows the RMSD metric plotted according to the level of tension forces. The RMSD was computed using the impedance data in the frequency band of 1–32 kHz. As observed from the figure, the RMSD value was increased linearly from 0% to 20.6% when the tension force rose from F0 = 9.81 kN to F4 = 49.05 kN. The increased value of the RMSD suggested the variation of tension forces. As discussed previously, the RMSD index reveals only the statistical change in impedance signatures that are not physically related to the mechanical properties of the host structure. Therefore, although the tension force change in an axial member can be effectively established by the RMSD metric, it is difficult to interpret the damage quantity using this statistical tool.

#### 4.3.3. Prediction of Tension Force Change in Axial Cylindrical Member

The tension force changes in the cylindrical structure were computed using Equation (5) with consideration of all three peak frequencies (Peaks 1–3), as listed in Table 6. Table 6 shows the predicted tension force changes for the case of complete load transfer from the axial member to the interface (i.e., the load transfer capability factor α = 1). From the table, it can be seen that when only Peak 1 was used to predict the tension force change, the error could be up to 26.5% of the inflicted value; the error when using only Peak 2 was relatively smaller, near 20%. The error corresponding to the use of Peak 3 was more significant, up to 31.5%. This means a single peak frequency could result in large errors for the tension force estimation. When all three impedance peaks were considered, the error was reduced significantly to 5% to 8%. The result demonstrated that the conceptual interface design is quite reasonable. The result also suggested that the more impedance peaks considered, the higher is the accuracy of the tension force estimation obtained.

#### 4.3.4. Effect of Load Transfer Capability of Wearable PZT Interface on Tension Force Estimation

**Figure 13:**
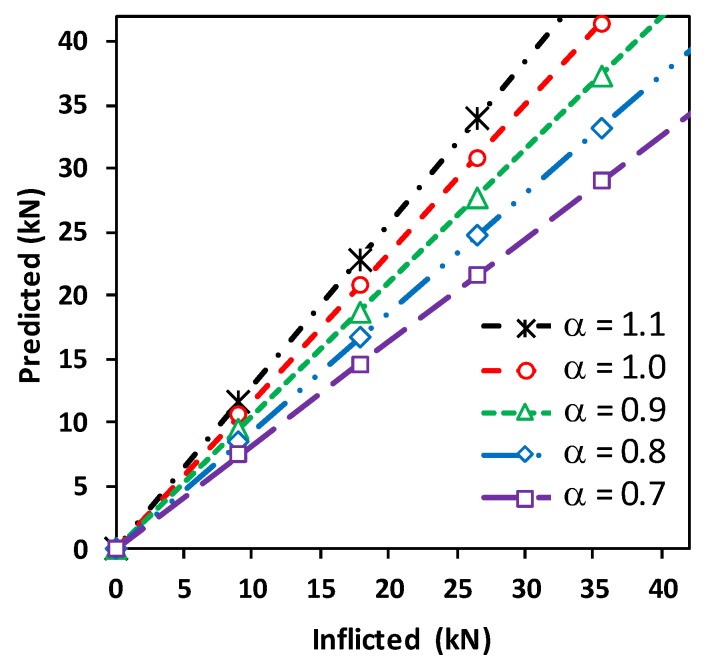
Prediction of tension force changes in axial cylindrical member: load transfer capability factor α = 0.7–1.1.

The effect of attachment condition of the wearable PZT interface on the accuracy of tension force estimation was examined by analyzing the load transfer capability factor α. The tension force changes in the test structure were computed for various values of α ranging from 0.7 to 1.1. As shown in Figure 13, the larger value of the load transfer factor resulted in the larger value of predicted tension force. It was found that the values close to 0.9 showed a good accuracy of predicted tension forces, and it was noted that α close to 1 shows a nearly complete load transfer (see Equation (4)). In the FE modeling, the PZT interface was perfectly bonded to the axially loaded cylindrical member, so the axial force was well transferred from the test structure to the PZT interface under tensioning. The numerical evaluation not only demonstrated the feasibility of the proposed method but also evidenced that the estimation of the load transfer capability factor was important for accurate axial load prediction. 

## 5. Experimental Evaluation on Lab-Scale Model of Cable Structure

### 5.1. Experimental Setup

**Figure 14:**
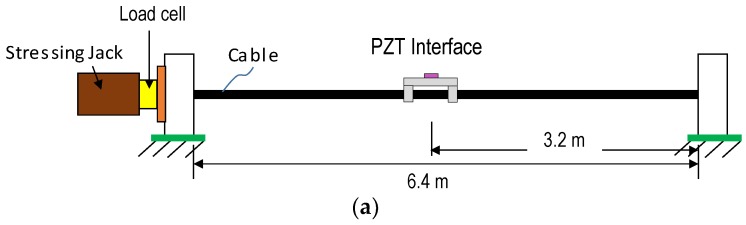

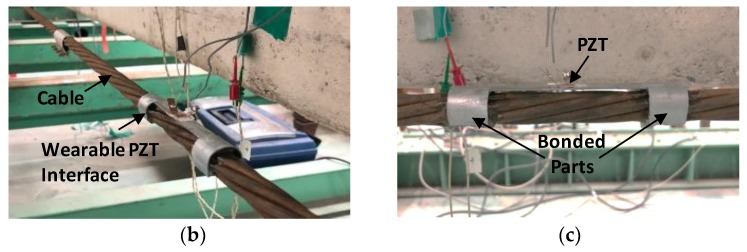
Experimental setup of lab-scale cable structure: (**a**) schematic of test setup; (**b**) cable equipped with wearable PZT interface; (**c**) attachment method.

The proposed axial force estimation method via the wearable PZT interface was evaluated on a lab-scale cable model. As shown in Figure 14a, the cable was anchored at the right end and tensioned at the left end using a stressing jack. A load cell was installed at the left end to measure the actual tension introduced into the cable. The cable comprised seven steel wires and was uncovered, as shown in Figure 14b. The steel cable structure had a length of 6.4 m, a nominal diameter of 15.2 mm, a nominal area of 138.7 mm^2^, a tensile strength of 260 kN, an elastic modulus of 190 GPa, and a unit mass of 1.37 kg/m.

By considering the dimensions of the cable, a wearable PZT interface was designed with the following geometric parameters: b_i_ × L_i_ × t_i_ = 10 × 80 × 1 mm for the flexible part, d_b_ × L_b_ × t_b_ = 17.2 × 10 × 1 for the hoop part, and b_a_ × l_a_ × t_a_ = 8 × 8 × 0.508 for the PZT patch (type PZT-5A). For easiness of fabrication, aluminum was used for the interface body. The wearable PZT interface was attached at the midpoint of the cable through the two outside contact parts by high-strength instant adhesive Loctite 401, as shown in Figure 14c. The PZT sensor was excited with a harmonic voltage 1V, and the EM impedance was measured in 0.2–15.5 kHz (766 swept points) using the impedance analyzer HIOKI-3532. 

Three levels of the tension force (i.e., F1–F3) were applied to the cable, as listed in Table 7. After the cable tension reached a desired force, impedance measurements were conducted. For each case of the tension force, four repeat impedance measurements were carried out. To avoid the temperature effect, room temperature was kept nearly constant with air conditioners during the tests.

### 5.2. Experimental Impedance Signatures of Wearable PZT Interface

**Figure 15:**
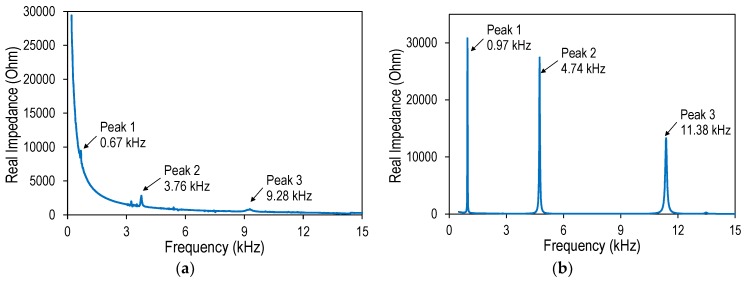
Experimental impedance signatures of wearable PZT interface under cable force F1: (**a**) experiment; (**b**) simulation.

The experimental impedance signatures of the wearable PZT interface under the tension force F1 are plotted in Figure 15a. Within the frequency band of 0.5–15 kHz, there were three distinct resonant peaks at 0.67 kHz (Peak 1), 3.76 kHz (Peak 2), and 9.28 kHz (Peak 3). Figure 15b shows the corresponding numerical impedance signatures simulated by COMSOL Multiphysics. Three distinct impedance peaks were also observed in the numerical signatures. Even though there were certain differences in the peak frequencies between the experiment and the simulation, the experimental impedance pattern was quite consistent with the simulation, suggesting the confidence of the impedance measurement.

**Figure 16:**
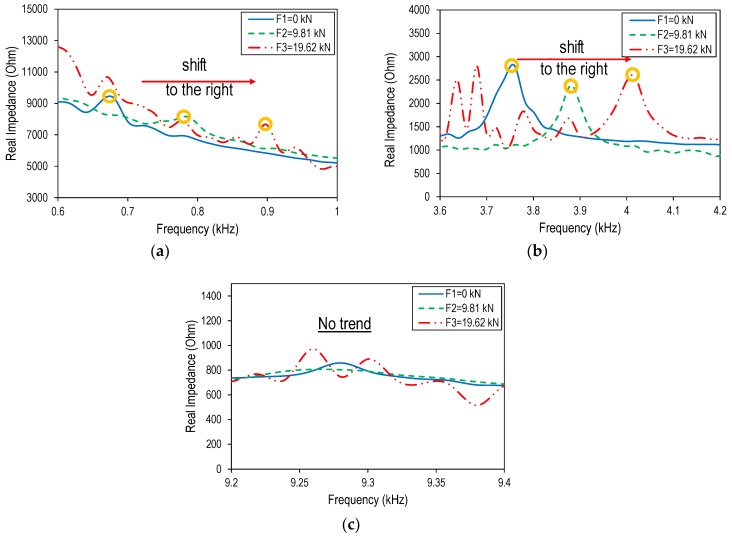
Shifts in experimental impedance peaks due to cable force changes: (**a**) Peak 1; (**b**) Peak 2; (**c**) Peak 3.

The three frequency bands containing the three impedance peaks (i.e., 0.6–1.0 kHz, 3.6–4.2 kHz, and 9.2–9.4 kHz) are shown in detail in Figure 16a–c, respectively. Under an increased cable force, the frequencies of Peak 1 and Peak 2 were sensitively shifted to the right, while the third peak had no apparent trend. Particularly, when the cable force was increased from F1 = 0 kN to F3 = 19.62 kN, the frequency of Peak 1 shifted from 0.67 kHz to 0.9 kHz (230 Hz increase), and the frequency of Peak 2 changed from 3.76 kHz–4.02 kHz (260 Hz increase). The increased peak frequencies suggested increments in the modal stiffness of the wearable PZT interface along with increased tension forces.

### 5.3. Prediction of Tension Force Changes in Lab-Scale Cable Structure

The cable force changes in the test structure were predicted using Equation (5) with consideration of the frequencies of Peaks 1–2 of the wearable PZT interface. Table 8 shows the predicted cable force changes for the case α = 1 (i.e., axial load perfectly transferred to the wearable PZT interface). From the table, it can be seen that when a single peak frequency or even multiple peak frequencies were used, the predicted cable force changes were only about half of the inflicted values. 

**Figure 17:**
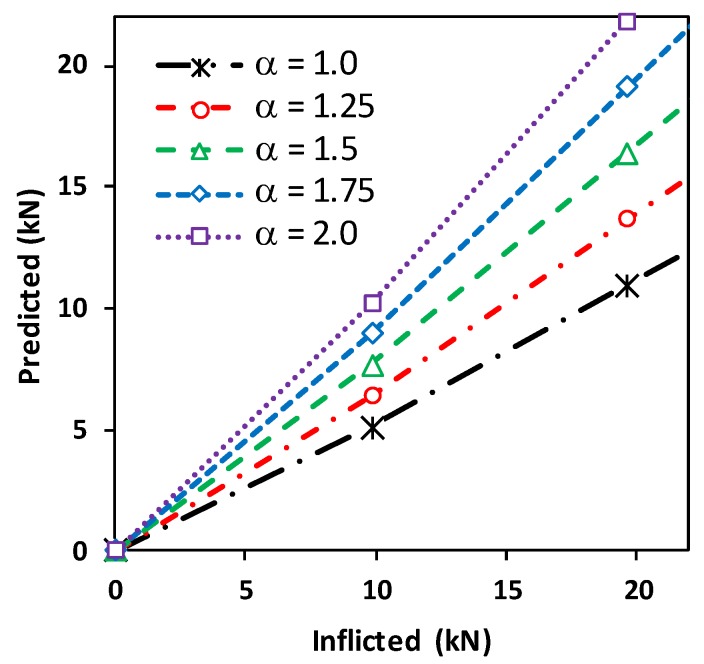
Prediction of tension force changes in the lab-scale cable structure: load transfer capability factor α = 1–2.

The effect of the load transfer capability of the wearable PZT interface on the tension force estimation was experimentally analyzed. Figure 17 shows the predicted tension force changes in the cable structure with various values of the load transfer capability factor α, ranging from 1 to 2. When the value of the load transfer factor was increased, the predicted cable force changes tended to approach the inflicted one. The load transfer capability factor of about 1.75 showed a good prediction of cable force changes. 

Compared with the FE modeling case, the experiment showed an increased value of the load transfer capability factor. In the experimental setup, the interface was directly attached to the cable, which comprised seven wires twisted into a helix. Thus, the hoop parts of the interface were not fully contacted with the cable when being installed. The reduced contact area caused decreased attachment strength of the interface, thus the value for the load transfer capability factor increased to 1.75. The obtained result demonstrated that the estimation of the load transfer capability of the wearable PZT interface was critical for an accurate axial force prediction. With a proper value of the load transfer capability factor, the proposed method could accurately estimate the tension load changes in an axial member. For real-world applications, the load transfer factor of the wearable PZT interface should be identified by preliminary tests.

## 6. Conclusions

This study proposes a novel wearable piezoelectric interface that can be used to monitor and quantitatively estimate the force changes in axial members. Firstly, an impedance-based force estimation method was presented for axially loaded members. The estimation was based on the relationship between the axial force level and the peak frequencies of impedance signatures, which were obtained from the wearable piezoelectric interface. Secondly, a prototype of the wearable piezoelectric interface was designed to be easily fitted into existing axial members. Finally, the feasibility of the proposed method was demonstrated by predicting axial load changes in the FE modeling of an axially loaded cylindrical member and a lab-scale model of a prestressed cable structure.

It was found that the estimation of the load transfer capability of the wearable PZT interface was an important factor for accurate axial force prediction. The numerical simulation evidenced that when the wearable PZT interface was completely bonded to the axial member (i.e., a nearly perfect load transfer condition), the proposed method could accurately predict axial force changes. In real situations where the wearable interface could receive a part of the axial load from the host structure, the load transfer capability factor should be appropriately determined for a high-fidelity prediction of the axial load.

While most of impedance-based SHM practices have utilized well-known damage metrics such as RMSD or CCD, which are only statistically related to the damage, this work explored a formula to physically describe the mechanical correlation between the axial load and the frequency shift in EM impedance signatures, thus opening up a new strategy to predict the axial load in axially loaded members. Despite the above positive findings, future studies are needed to optimize the geometric parameters of the wearable PZT interface and experimentally validate the practicality of the proposed method on in-situ structures. In addition, the hoop parts of the interface should be adjusted to fit with spiral fluted surface of target structures in order to enhance the load transfer capability.

## Figures and Tables

**Table 1:** Nondimensional natural frequencies dependent on boundary conditions.

Mode	Nondimensional Natural Frequency C_n_	
	Fixed-Fixed	Pinned-Pinned
1	22.3733	π^2^
2	61.6728	4π^2^
3	120.9034	9π^2^
4	199.8594	16π^2^
5	298.5555	25π^2^

**Table 2:** Mechanical properties of the steel interface body.

Elastic Modulus E (GPa)	Mass Density ρ (kg/m^3^)	Poisson’s Ratio υ	Damping Loss Factor η
200	7850	0.3	0.02

**Table 3:** Piezoelectric properties of the PZT patch.

Elastic Modulus Y^E^_11_ (N/m^2^)	Mass Density ρ (kg/m^3^)	Coupling Constant d_31_ (m/V)	Dielectric Constant ε^T^_33_ (Farads/m)	Damping Loss Factor η	Dielectric Loss Factor δ
6.1 × 10^10^	7650	−1.71 × 10^−10^	1.53 × 10^−8^	0.0125	0.015

**Table 4:** Simulation cases of tension forces in axial cylindrical member.

Case	Inflicted Tension Force (kN)	
	F	ΔF
F0	9.81	0
F1	19.62	9.81
F2	29.43	19.62
F3	39.24	29.43
F4	49.05	39.24

**Table 5:** Natural frequencies (kHz) of the wearable PZT interface under tension force changes.

Case	Peak 1			Peak 2			Peak 3		
	Imp. Analysis	F-F Model	P-P Model	Imp. Analysis	F-F Model	P-P Model	Imp. Analysis	F-F Model	P-P Model
F0	2.25	2.28	1.23	11.78	11.43	8.60	28.61	27.91	23.25
F1	2.39	2.47	1.49	12.02	11.63	8.95	28.88	28.12	23.60
F2	2.53	2.64	1.70	12.25	11.84	9.29	29.12	28.33	23.95
F3	2.65	2.80	1.89	12.48	12.04	9.61	29.36	28.54	24.29
F4	2.77	2.96	2.07	12.71	12.24	9.92	29.62	28.74	24.63

**Table 6:** Prediction of tension force changes (kN) in the finite element (FE) model of axial cylindrical member.

Infliction		Prediction (α = 1)							
		Using Peak 1		Using Peak 2		Using Peak 3		Using Peaks 1–3	
Case	ΔF	ΔF	Error	ΔF	Error	ΔF	Error	ΔF	Error
F0	0	0	0	0	0	0	0	0	0
F1	9.81	7.21	26.50%	11.72	19.47%	12.90	31.50%	10.61	8.15%
F2	19.62	14.84	24.36%	23.17	18.09%	24.46	24.67%	20.82	6.13%
F3	29.43	21.73	26.16%	34.84	18.38%	36.13	22.77%	30.90	4.99%
F4	39.24	28.94	26.25%	46.73	19.09%	48.87	24.54%	41.51	5.79%

**Table 7:** Test cases of tension forces in the cable structure.

Case	Inflicted Cable Force (kN)	
	F	ΔF
F1	0	0
F2	9.81	9.81
F3	19.62	19.62

**Table 8:** Prediction of tension force changes (kN) in lab-scale cable structure.

Infliction		Prediction (α = 1)					
		Using Peak 1		Using Peak 2		Using Peaks 1–2	
Case	ΔF	ΔF	Error	ΔF	Error	ΔF	Error
F1	0	0	0	0	0	0	0
F2	9.81	4.99	49.13%	5.21	46.89%	5.10	48.01%
F3	19.62	11.00	43.93%	10.86	44.65%	10.93	44.29%
